# In dogs with snakebites does the use of antimicrobials compared to no antimicrobials reduce incidence of wound infection?

**DOI:** 10.18849/ve.v8i3.513

**Published:** 2023-07-28

**Authors:** Megan Ballman+, Davide Messina+

**Keywords:** DOG, CANINE, SNAKEBITE, SNAKE, ENVENOMATION, ANTIBIOTIC, ANTIBIOTIC RESISTANCE, ABR, ANTIMICROBIAL, ANTIMICROBIAL RESISTANCE, AMR, ONE HEALTH

## Abstract

**PICO question:**

In dogs with snakebites does the use of antimicrobials compared to no antimicrobials reduce incidence of wound infection?

**Clinical bottom line:**

**Category of research:**

Treatment.

**Number and type of study designs reviewed:**

Seven case series.

**Strength of evidence:**

Weak.

**Outcomes reported:**

Three studies looked at the incidence of wound infection in envenomated dogs, three compared antimicrobial use to mortality and one compared other outcomes, such as time in hospital. The incidence of wound infection was low in the studies and overall antimicrobials seemed to have no significant effect on outcomes such as survival or wound infection. Therefore, the routine use of antimicrobials for snakebite treatment is not supported by the results, however further studies are required to provide conclusive evidence.

**Conclusion:**

There is currently insufficient evidence from literature to either support or reject the use of antimicrobials in the treatment of snakebites.

## How to apply this evidence in practice

The application of evidence into practice should take into account multiple factors, not limited to: individual clinical expertise, patient’s circumstances and owners’ values, country, location or clinic where you work, the individual case in front of you, the availability of therapies and resources.

Knowledge Summaries are a resource to help reinforce or inform decision making. They do not override the responsibility or judgement of the practitioner to do what is best for the animal in their care.

## Clinical scenario

A client has brought in a dog that has a suspected snakebite. You are aware that infection in the wound is possible due to bacteria present on snake fangs, but you want to only prescribe antimicrobials when they are necessary. You want to know whether there is evidence that the use of antimicrobials for this case will improve clinical outcome by reducing incidence of wound infection compared to no antimicrobial intervention.

Snakebites have long been treated with antivenin, but the therapeutic approach in terms of ideal supportive care remains up for debate. Snakes are known to harbour bacteria in their oral cavity, including *Salmonella*, *Pseudomonas aeruginosa, Proteus spp*., coagulase-negative *Staphylococcus spp., Clostridium spp*., and *Bacteroides fragilis *(Peterson, 2006). Consequently, many veterinary references recommend the use of antimicrobials following envenomation to prevent wound contamination. Comparable research on this topic is lacking in veterinary medicine, nevertheless with the increasing issue of antimicrobial resistance, it is ever more vital for veterinarians to dispense antimicrobials only when they are needed. It is therefore important to consider whether the use of antimicrobials in the treatment of snakebites reduces the risk of wound infection, and therefore whether they are a necessary part of the therapeutic treatment.

## The evidence

There is a lack of evidence available that supports the PICO question due to the existence of very few papers that analyse the use of antimicrobials, which are referred to as antibiotics in some papers. At the time of writing there were no studies directly comparing antimicrobial treatment to no antimicrobial treatment. Two studies (Lervik et al., 2010; and Carr & Schultz, 2015) were identified that look at the incidence of wound infection following a snakebite. Although no statistical analysis was performed, Pritchard et al. (2014) reported incidence of infection following snakebite of the dogs in their paper so was also included. The papers by McCown et al. (2009) and Kim et al. (2022) were deemed relevant due to the evaluation of the clinical outcome of survival following antimicrobial administration. Similarly, Witsil et al. (2015) presented data about antimicrobials and rate of mortality, while Hackett et al. (2002) compared outcomes such as clinicopathological data, treatment costs and days in hospital, to use of antimicrobials.

Overall, current study limitations provide insufficient evidence to support or reject the use of antimicrobials. Further randomised controlled trials (RCT) that directly compare wound infection to antimicrobial administration would greatly help in providing a more reliable proof for this PICO.

## Summary of the evidence

**Carr & Schultz (2015):** 

Population:	Dogs that were presented following rattlesnake envenomation, at a 24-hour referral hospital in Murrieta, California, between March 2012 and May 2013. Inclusion criteria: Received no antimicrobials. Witnessed rattlesnake envenomations. Highly suspected envenomations (e.g. rattle was heard, patient yelped and consistent bite wounds evident). Suspected envenomations with bite marks, swelling and echinocyte tests that were >90% positive. Exclusion criteria: Were placed on antimicrobials by referring veterinarian prior to transfer. Were initiated on antimicrobials during hospitalisation for conditions unrelated to envenomation. Death or euthanasia within 24 hours of presentation. Lost to follow-up conducted within 2 weeks by phone or direct inspection of wound.
Sample size:	102 dogs.
Intervention details:	Most (82%) were treated with: intravenous (IV) crystalloid fluids; hydromorphone; antivenom most received crotalidae polyvalent antivenom or received crotalidae polyvalent immune Fab (n = 1); release within 24 hours. Severe cases (23.5%): Diagnosis based on clinical signs combined with either a decreased platelet count, increased prothrombin time, or both, consistent with venom-induced coagulopathy (VIC). Developed VIC was hospitalised for 2 days (n = 1): received crotalidae polyvalent immune fab (n = 1). Developed infection (abscess to thoracic limb) suspected to be secondary to compartment syndrome (n = 1): empirical therapy instituted using cefazolin 10 mg/kg IV every 8 hours; aerobic and anaerobic bacterial culture and sensitivity obtained from the abscess; therapy continued with cephalexin 22 mg/kg PO every 12 hours for 14 days; abscess surgically debrided and managed with wet-to-dry bandages until the wound could be closed primarily. Showed signs of flaccid paralysis and muscle fasciculations consistent with neurotoxic envenomation (n = 1): hospitalised for 3 days. Owner declined hospitalisation, (18/102): defined as ‘outpatient’ – hospitalisation for ≤ 4 hours; received antivenin and narcotic analgesics (n = 12); declined all treatment apart from analgesics (n = 6).
Study design:	Prospective observational case series.
Outcome studied:	Location of bite and head strike. Treatment used. Mortality. Follow-up: direct examination of wound within 2 weeks or; telephone contact with questions regarding: wound healing; signs of infection; need for follow-up care. Incidence of wound infection. In one case with an abscess aerobic and anaerobic bacterial culture and sensitivity was obtained.
Main Findings (relevant to PICO question)	Three patients died shortly after presentation from conditions that were clearly not antimicrobial responsive: mortality rate = 2%. Of 102 cases, only one developed an infection, that was secondary to suspected compartment syndrome and development of an abscess to the thoracic limb. *Staphylococcus aureus* and *Pasteurella* species were cultured from one case, the anaerobic culture was negative: more consistent with skin contamination of the victim rather than oral flora of the snake.
Limitations:	Power of study not calculated. Loss to follow-up (n=4). Inadequate follow-up techniques as phone calls replaced physical inspection of wound in most cases. Cannot confirm subclinical infection develops. Did not use a severity scoring system to identify wounds that could potentially become necrotic. Treatment and monitoring protocols not standardised.

**Hackett et al. (2002):** 

Population:	Dogs treated for snakebite at the Colorado State University Veterinary Teaching Hospital during a 10-year period (1989–1998). Inclusion criteria: contact with a rattlesnake was witnessed or; compatible clinical signs of severe swelling around paired puncture wounds. Exclusion criteria: Any doubt in the medical record of the diagnosis of rattlesnake envenomation.
Sample size:	100 dogs.
Intervention details:	Hospitalised (n = 94) or outpatient treatment (n = 6). Intravenous (IV) fluid therapy consisting of crystalloid fluids (n = 84). One unit fresh whole blood (n = 3). One unit fresh frozen plasma (n = 1). Heparin (n = 10). Narcotic analgesic drugs (n = 15): morphine, oxymorphone and fentanyl. Ketoprofen 1.5 mg/kg (n = 1). Antivenin (n = 23). Glucocorticoids (dexamethasone sodium phosphate) (n = 87): alone (no antihistamine) n = 32; with antihistamine n = 55. Antihistamine (diphenhydramine or pyrilamine) (n = 59): alone (no glucocorticoid) n = 4; with glucocorticoid n = 55. Antimicrobial (n = 87): ampicillin alone (n = 54); ampicillin and enrofloxacin (n = 5); amoxicillin alone (n = 9); amoxicillin and enrofloxacin (n = 5); cefazolin (n = 3); cefoxitin (n = 2); gentamicin (n = 1); trimethoprim sulfamethoxazole (n = 1).
Study design:	Retrospective case series.
Outcome studied:	Patient signalment. Geographic location of the snakebite incident. Date and time of envenomation. Time to reach the hospital. Anatomic location of the bite. Clinical laboratory abnormalities. Treatments. Costs of all treatments. Duration of hospitalisation. Complications and clinical outcome.
Main Findings (relevant to PICO question)	Multiple linear regression was performed to model each response (logarithm of cell counts, cost, or days in hospital) as a function of: variables determined at initial evaluation (age, weight, sex, PCV, and anatomic location of the bite); treatments received (antimicrobials, glucocorticoids, antihistamines, analgesics, and antivenin). For all comparisons, significance was set at a value of P ≤ 0.05 There was no significant association between antimicrobial treatment and any outcome variables. No wound related complications (infections, abscessation, necrosis) were identified in the medical records.
Limitations:	Power of study not calculated. Retrospective, so limited evidentiary value. Large variation in treatment protocols, including type of antimicrobial, and lack of detail with some interventions. The single reported figures for the antimicrobials add up to 80 and not 87 dogs. The study gave no reason for this discrepancy. Follow-up of patients beyond discharge was not performed, so possible long-term complications were not identified. Hospital stay ranged from <1 day to 5 days so variety in time monitored for complications. Some cases were treated as outpatients were not able to be monitored by trained clinicians. Small numbers of dogs that did not receive antimicrobials to determine comparison. Only outcomes described were duration of hospital stay and mortality so limited analysis of longer-term outcomes. No description of how follow-up was carried out.

**Kim et al. (2022):** 

Population:	Dogs admitted with snakebites to the Kyungpook National Veterinary Medical Teaching Hospital, Haemaru Animal Referral Hospital, and Daegu Animal Medical Center from June 2008 to July 2021. Inclusion criteria: dogs in which two fang marks observed at the wound site. Exclusion criteria: dogs with bite wounds of unclear origin.
Sample size:	70 dogs.
Intervention details:	Antivenom (n = 53). Analgesic: tramadol (n = 29); hydromorphone (n = 10); tramadol, lidocaine, ketamine (n = 8); fentanyl (n = 2); butorphanol (n = 1). Fluids: normal saline (n = 29); plasma-lyte (n = 12); Hartmann’s solution (n = 6); 5% dextrose water (n = 1); 5% dextrose water (n = 2). Chlorpheniramine (n = 51). Glucocorticoids (n = 32). Antibiotics (n = 62).
Study design:	Retrospective case series.
Outcome studied:	Signalment. History including witnessed snakebite and time since the snakebite. Clinical signs and physical examination records on presentation. Routine test results: complete blood count, serum chemical profile, coagulation profile, blood smear evaluation. Treatment received. Length of hospitalisation. Outcomes: ‘Survivors’ – discharged alive or referred to another hospital; ‘Non-survivors’ – died.
Main Findings (relevant to PICO question)	The χ2 test was performed to compare categorical data between survivors and non-survivors. Statistical significance was set at a p value of < 0.05: among 42 hospitalised dogs with follow-up data, 37 (88%) survived to discharge and 5 (12%) died; the administration of antibiotics was associated with increased survival (P = 0.030); the study could not reveal any benefit or detriment of the use of antibiotics; the study revealed an association of antibiotic use with survival but could not demonstrate that antibiotic use affected survival.
Limitations:	No information regarding how many dogs survived when treated with antibiotics. Power of study not calculated. Retrospective, so limited evidentiary value. Incomplete medical records. Many dogs in the study presented to the hospital only for antivenom treatment; thus, the possibility of follow-up and assessment of prognosis was limited. Comparing outcomes is difficult without standardised treatment and variation in clinicians. Only 42 dogs had follow-up data so cannot assess long-term outcome and means there is a low sample size for statistical analysis. Could not confirm snake species so this variation may be confounding. Complications could not be evaluated properly because serial laboratory examinations were performed only in a small number of dogs.

**Lervik et al. (2010):** 

Population:	Dogs bitten by the *Vipera berus* presented at the Södra Djursjukhuset in Stockholm or the University Animal Hospital in Uppsala during the period of April to August 2006. Inclusion criteria: strong suspicion of viper bite at the time of presentation based on information from the dog owner (bite was witnessed, or viper seen close to dog); and / or; clinical signs of viper bite (e.g., lethargy or swelling in the suspected bite area). Exclusion criteria: ongoing treatment with glucocorticoids for other conditions; known history of liver disease.
Sample size:	53 dogs.
Intervention details:	Fluid therapy consisting of crystalloid fluid at 40–60 ml/kg/h to 40–60 ml/kg/day (n = 53). Fluid therapy consisting of colloid fluids at 5–20 ml/kg/10min to 0.8 ml/kg/day (n = 28). Glucocorticoids: none (n = 31); pre-admission from referring (n = 7): 1–0.25 mg/kg betamethasone per os, once (n = 5); 6 mg/kg prednisolone injection, once (n = 1); 1 mg/kg prednisolone injection per os, once (n = 1); at admittance (n = 16) (two dogs had additionally been treated by owner): 1–2 mg/kg prednisolone injection, once (n = 11); 2–2 mg/kg prednisone injection, for 2–5 days (n = 2); 1–1.25 mg/kg prednisolone per os, once (n = 3). Analgesics (n = 36): buprenorphine, methadone hydrochloride or transdermal fentanyl for 1–6 days – no drug or dosage specified. Antibiotics (n = 10): no drug or dosage specified. Antiarrhythmic drug (n = 2).
Study design:	Prospective case series.
Outcome studied:	Blood samples taken at four different times: arrival (n = 53); 24 hours (n = 52); day 4–10 (n = 46); day 9–23 (n = 33).Serum harvested and frozen until a time point 1–5 months after presentation. Variables recorded: Mental status – alert, or slightly, moderately or severely depressed. Degree of swelling – minor, moderate or severe. Serum biochemistry (n = 34): alanine aminotransferase (ALT); alkaline phosphatase (ALP); bile acids; glutamate dehydrogenase (GLDH); creatine kinase (CK); creatinine. Cardiac variables on auscultation. Electrocardiogram (ECG) of dogs that had detected arrhythmias (n = 5).
Main Findings (relevant to PICO question)	No clinical signs of infection in any dog in the study, even though only 10/53 (19%) were treated with antibiotics.
Limitations:	One of the reported values for fluid therapy consisting of colloid fluids (i.e., 0.8 ml/kg/day) seems very small so may not be accurately reported. The reported figures for the baseline group (i.e., the dogs not receiving glucocorticoids, n = 31) and for the exposed group (i.e., the dogs receiving glucocorticoids, n = 7 and n = 16) add up to 54 and not 53 dogs. The study gave no reason for this discrepancy. Time points for follow-up were variable with a wide range. Small sample size of antibiotic treated dogs reduced statistical power. Twenty dogs lost to follow-up and could only collect serum from 34/53 dogs as 19 were not available for all the timepoints, resulting in a reduced sample size. Some dogs had concurrent diseases which may have been confounding. Variation in veterinarian that examined and non-standardised measurements of variables, such as degree of swelling, meaning reliability of clinical ratings is uncertain. Large variation in treatment protocols, with different dosages, routes and measuring timepoints. Concluded that no clinical signs of infection were found but these signs were not described so cannot be sure if the diagnoses were correct. No basal value obtained for biochemical parameters used in statistical analysis so difficult to conclude that the changes were significant. Lack of, and poorly reported, statistical analysis so unknown if outcomes are significant.

**McCown et al. (2009):** 

Population:	Dogs presented to a private referral centre and university teaching hospital in the United States from 1988–2006. Inclusion criteria: dogs with puncture wounds combined with presence of clinical signs consistent with crotalid envenomation (including swelling, haemorrhage, or ecchymoses at the site of the bite); treatment with polyvalent IgG antivenin; absence of prior history of envenomation; complete medical record (including signalment, body weight, amount of antivenin administered, and outcome). Exclusion criteria: treatment with fab antivenin; incomplete medical record.
Sample size:	218 dogs.
Intervention details:	Intravenous (IV) fluids (n = 213). Diphenhydramine (n = 150): median dose 2.0 mg/kg; dose not recorded (n = 24). Corticosteroids (n = 35): dexamethasone (n = 23); dexamethasone sodium phosphate (n = 2); prednisolone sodium sucinate (n = 7); methylprednisolone sodium succinate (n = 3). Antimicrobials (n = 196): β-lactam antimicrobials alone (n = 167); azole antimicrobial alone (n = 1); more than one type: including β-lactam, azole, fluoroquinolone, and other (n = 28). Epinephrine (n = 6). Heparin (n = 8). Blood products (n = 26): plasma (n = 15); whole blood (n = 9); packed red blood cell transfusions (n = 2); infusion of bovine haemoglobin (n = 1). Opioid analgesics (n = 178).
Study design:	Retrospective case series.
Outcome studied:	Patient signalment: breed, sex, age, weight. Anatomic area of bite(s) and number of bite(s). Species of snake involved in bite (if known). Physical examination findings at time of presentation: rectal temperature, pulse rate, respiratory rate, and the presence of swelling, haemorrhage, or petechia / ecchymoses; mentation (bright, alert, and responsive; quiet, alert, and responsive; quiet, depressed, but responsive; or other). Clinicopathological data at time of presentation: packed cell volume (PCV), total plasma protein, platelet count, prothrombin time (PT), and partial thromboplastin time (PTT). Treatment received. Number of vials of antivenin administered. Complications of antivenin therapy. Length of hospitalisation. Cost of treatment. Clinical outcome (survival vs death): defined as either natural cardiorespiratory arrest or euthanasia.
Main Findings (relevant to PICO question)	Penalised logistic regression was used to determine the effect of covariates, looking at survivors vs non-survivors, including administration of antimicrobials. 9/218 (4.1%) died: no data on which of these had received antimicrobials. 196/218 (90%) were administered antimicrobials: 167 were administered β-lactam alone; one was administered an azole antibiotic alone; 28 were administered more than one type of antibiotic (including β-lactam, azole, fluoroquinolone, and other). Fluoroquinolone administration was associated with an increased likelihood of survival (P = 0.046). Not significantly related to survival: β-lactam antimicrobials; azole antimicrobials; other antimicrobials. Low mortality rate (n=9).
Limitations:	Retrospective, so limited evidentiary value. Time period of 18 years, so treatment recommendations have changed. Insufficient numbers to achieve adequate power. Variety of snake species measured, the bites of which may cause different clinical outcomes. The distribution of cases between the two locations is not described and is therefore a source of potential bias due to possible differences in protocols. No uniform severity scoring, so patients with a broad range of clinical presentations were being compared. Lack of uniform treating regime as many clinicians involved. Treatment confounded by cost considerations, experience of clinician and reliance on anecdotal reports. Several variables were documented in too few dogs to allow statistical evaluation. Did not consider interactions of the covariates in any statistical analysis models. The dosage and administration for antimicrobials was not recorded but could be confounding. The prevalence of wound infection was not assessed. No information regarding how many dogs treated with antibiotics survived Low mortality rate (n = 9), reliable conclusions cannot be made.

**Pritchard et al. (2014):** 

Population:	Dogs presented to North Carolina State University Veterinary Teaching Hospital from 2004 to 2011. Inclusion criteria: dogs residing in Wake County that presented within 12 hours of a suspected copperhead bite; ‘confirmed’ cases if owners either saw the copper-head bite the dog or found the dog with fang marks and a snake nearby; ‘suspected’ cases were categorised as those in which an animal had at least one fang mark, clinical signs consistent with snakebite, and a history compatible with the possibility of snakebite. Exclusion criteria: had been previously presented for medical treatment at another facility for this presentation.
Sample size:	52 dogs.
Intervention details:	Antimicrobials while hospitalised (n = 35). ampicillin Na / sulbactam Na (n = 25); amoxicillin trihydrate / clavulanate potassium (n = 6); cefazolin (n = 2); ciprofloxacin (n = 2). With antimicrobials sent home (n = 48): amoxicillin trihydrate / clavulanate potassium (n = 40); cephalexin (n = 6); ciprofloxacin (n = 6). Glucocorticoids – dexamethasone (n = 9). Analgesia in hospital (n = 48): hydromorphone (n = 20); fentanyl (n = 18); buprenorphine (n = 9); tramadol (n = 4); carprofen (n = 2). With analgesia sent home (n = 49): tramadol (n = 41); carprofen (n = 10); fentanyl (n = 5); meloxicam (n = 5); buprenorphine (n = 1). Diphenhydramine (n = 11). Famotidine (n = 3). Intravenous (IV) fluids (n = 33).
Study design:	Retrospective case series.
Outcome studied:	Signalment. Presenting complaint. ‘Confirmed’ vs ‘suspected’ cases. Date of presentation. Time from presentation to suspected envenomation. Vital parameters (temperature, pulse, and respiration) and weight. Clinical signs as described by the attending clinician. Pain scoring. Clinicopathological data: packed cell volume, total solids, blood glucose, complete blood count, serum biochemical analysis, coagulation panel. Treatments administered including those prescribed to give at home. Bill total for the visit. Total hospitalisation time. Whether the patients either improved or worsened during their hospitalisation time given the information provided by the record. Follow-up phone or e-mail to owner: improvement at home after discharge; if the dog required follow-up medical care at another facility; if the dog developed evidence of bite site necrosis.
Main Findings (relevant to PICO question)	Tissue necrosis at the bite site was reported by 3/27 owners (11%): Of those, only one required follow-up care at another veterinary facility and resolved with antibiotic treatment without the need for surgical debridement. Self-limiting infection at the bite site was reported by 1/27 owners (4%) that did not require either follow-up treatment at an additional veterinary facility or changes in treatment protocol. No dog bitten by a copperhead was re-presented to the study authors’ emergency facility for snakebite-associated abscess formation. All dogs survived to discharge.
Limitations:	We only know about dogs’ survival to discharge and that owner’s phone or e-mail follow-up survey response was only available for 27/52 dogs (52%), so limited; therefore, we do not know how many were lost to follow-up as the article does not provide this information. Power of study not calculated. Retrospective, so limited evidentiary value. No statistical analysis performed. Information is limited to that which was recorded in the medical record at the time of hospitalisation. Comparing outcomes is difficult without standardised treatment and variation in clinicians. Could not confirm snake species so this variation may be confounding. Follow-up assessment of infection at the bite site relied on owner assessment so is less reliable. No indication of whether the dogs that had complications related to infection had received antibiotics, so cannot determine this significance. Interventions included ‘treated with antimicrobials in hospital’ and ‘sent home with antimicrobials’ but there was no indication of the overlap of these groups, though it could be inferred that at least 48 dogs had some antimicrobial treatment (those sent home). Outcome of ‘whether the patients either improved or worsened during their hospitalisation time given the information provided by the record’ is subjective. Median duration of hospitalisation in dogs was 14.8 hours so no extended period of observation for complications by clinicians.

**Witsil et al. (2015):** 

Population:	Dogs from six veterinary emergency centres in Maricopa County, Arizona between 2010 and 2012. Inclusion criteria: dogs with witnessed rattlesnake envenomation or clinical signs consisted with envenomation (rapid swelling, pain, puncture wounds) and; dog had access to rattlesnake(s) at time of onset of clinical signs.
Sample size:	272 dogs.
Intervention details:	Antivenom: antivenin *Crotalus durissus* and *Bothrops asper* (‘F(ab′)2 AV’) given at the five hospitals: reconstituted using 10 mL sterile 0.9% saline, bacteriostatic water, Lactated Ringer’s solution, or Plasma-Lyte fluid. antivenin (Crotalidae) polyvalent (ACP) (‘IgG AV’) given primarily at referring clinics prior to transport: reconstitution similar to F(ab’)2 AV, but precise methods for administration were not uniformly recorded by referring clinics and could not be described. Rattlesnake vaccine (n = 15). Antimicrobials (n = 64): β-lactams (n = 48); no antimicrobials (n = 207); unknown antimicrobial (n = 1). Glucocorticoids: no number stated. Diphenhydramine: no number stated.
Study design:	Retrospective case series.
Outcome studied:	Patient demographics. Age of patient. Location of bite. Time to presentation. Month of envenomation. Prior rattlesnake vaccination. Canine snakebite severity score (cSSS) based on the validate human version summation of Bite Factor scores (swelling, ecchymosis, pain, and drainage) is given a score of either 0 (if absent) or 1 (if noted) and; clinical sign scores (1 point given to each clinical sign noted). Treatments received: number of vials of antivenom used; relationship between body weight and vials used; type of antibiotic. Length of stay in the hospital. Survival.
Main Findings (relevant to PICO question)	The data were converted into ranks and parametric statistical analyses were performed: 24% cases (n = 64) received antibiotics; mortality was 8/272 (died n = 4, euthanised n = 4): three fatal cases had received antibiotics; five had not received antibiotics. Significantly longer duration of hospital stay was found in patients that received antibiotics (P = 0.0004).
Limitations:	No recording or verification of the envenoming crotaline species, which may have been helpful in assessing the outcome. Canine snakebite severity score scale has not been validated and individual patient variables are not accounted for, meaning the scale should be narrowly interpreted. Challenge of grading animals due to lack of verbal descriptive feedback. Cost to owner of measurements the components of cSSS is confounding as this limited the diagnostics in some cases. Treatment was prioritised over acquiring laboratory measurements when finances were a limiting factor. Retrospective. Administration of treatment depended on severity of clinical signs, patient tolerance of infusion, and clinician preference. Low number of fatalities prohibited statistical comparison of the data on survival. Specific clinical changes or comorbidities leading up to cardiopulmonary arrest or factoring into decisions to euthanise were not recorded. Follow-up of patients beyond discharge was not performed, so possible complications were not identified.

## Appraisal, application and reflection

The literature search performed by the author found seven papers which partially addressed the PICO question. Apart from Carr & Shultz (2015) there have been no studies so far evaluating the use of prophylactic antimicrobials in envenomated animals. In the seven papers sample size calculation was lacking and statistical analysis was sometimes poorly reported, thus hindering interpretation of significance. The studies are limited due to a lack of standardised treatment and monitoring protocols, and no consistent assessment regarding severity of envenomation and wound infection. There is wide variation in supportive treatments given in the literature, thus inconsistent protocols were described, which makes direct comparison of the effect of antimicrobials more difficult to assess. Notably, steroids were given to a number of dogs in these studies and this may have impacted both the incidence of infection and the response to antimicrobials in these cases. It is also likely that there is variability in clinical outcomes, due to factors such as location of the bite and time to treatment. Finally, individual variation in venom profiles has been proven between specimens of the *Vipera berus*, manifesting in a wide range of clinical features (Malina et al., 2017). Therefore, another confounding factor is the differing venom phenotypes between the range of snake species reported in these studies.

Firstly, cases of suspected acute rattlesnake envenomation that had received no antimicrobial treatment were enrolled in the study by Carr & Schultz (2015). Within 2 weeks of presentation they were evaluated regarding wound healing, signs of infection and need for follow-up care by direct examination of the wound, or telephone contact when this was not possible. Of the 102 cases, only one developed an infection; an abscess to the thoracic limb that was secondary to suspected compartment syndrome. The abscess was cultured and *Pasteurella* and *Staphylococcus *species were found, but these are consistent with skin contaminants, as opposed to the oral flora of the snake (Carr & Schultz, 2015). This supports the view that incidence of infection in envenomation is low and therefore prophylactic antimicrobials need not be part of a treatment protocol. However, it could not be confirmed that subclinical infections did not develop as there is no accepted way to rule out a sub-clinical infection  other than culture of wounds that had obvious abscessation and necrosis. In addition, patients were lost to follow-up which may have caused selection bias, and there were cases that were included in analysis even though direct examination was not possible. Furthermore, there was evident bias due to a large variation in treatment protocols from different veterinarians and non-standardised measurements of variables such as clinical signs.

Hackett et al. (2002) evaluated 100 cases of snakebite envenomation in dogs at a hospital over a 10-year period. Multiple linear regression was performed to model outcomes of cell counts, cost and days in hospital as a function of variables such as treatment with antimicrobials, with significance set as P ≤ 0.05. They found no significant association between antimicrobial treatment and any outcome variable. Nevertheless, this method of statistical analysis is limited by low population (n = 100), possible incomplete data and variables may not be independent. In total 87/100 (87%) of dogs received antimicrobials and the authors noted that no wound related complications (infections, abscessation, necrosis) were identified in the medical records which could support use of antimicrobials. But as so few dogs did not receive antimicrobials it is difficult to conclude this due to lack of data for comparison between groups. Also, the lack of follow-up after discharge means long-term complications have not been identified, so infections that developed later may have been missed.

Records of dogs admitted with snakebites to three hospitals between 2008–2021 were reviewed by Kim et al. (2022) in their retrospective case series. Part of their analysis included performing a χ2 test to compare categorical data between survivors and non-survivors. The administration of antimicrobials was associated with increased survival (P = 0.030) which may therefore support use of antimicrobials in these cases. However, Kim et al. (2022) recognised that though the study revealed this statistical association, they could not demonstrate that antimicrobial use affected survival. Although 72 cases were included in the study, only 42 had follow-up data to be able to be included in this statistical analysis, meaning a low population is even more limiting. Furthermore, assessment of long-term prognosis was impossible due to incomplete medical records and lack of follow-up. Limitations of this retrospective study include lack of follow-up, no proof of cause-effect, no treatment standardisation and lack of a control.

In the paper by Lervik et al. (2010), 53 dogs that presented with a strong suspicion of viper bite and / or clinical signs of viper bite were included in their analysis. No mortalities occurred, however the mortality rate of dogs bitten by the European adder has been reported to be at 4.6% (Sutton et al., 2011). Therefore, it is possible that the lack of observed difference found in this study is due to small sample size rather than lack of effect. Of the population, 10/53 (19%) were given antimicrobials, but clinical signs related to infection were not seen in any of the cases. This could suggest that the administration of antimicrobials does not have an effect on the incidence of wound infection. This finding is consistent with the study by Sutton et al. (2011) where 235/422 (56%) of cases received antimicrobials, yet infection was reported in none. Nevertheless, despite this conclusion by Lervik et al. (2010), the measurements for clinical signs of infection were not described so it cannot be certain that the diagnoses were correct. The lack of objectivity in the study design, such as treatments used and time points for follow-up, along with the confounding effects of concurrent diseases, means that conclusions of the study must be interpreted with caution. In fact, there was a lack of statistical analysis, and where there was it is poorly reported, so it is unproven whether the outcomes of this study are significant.

Although the study by McCown et al. (2009) does not directly address the PICO, it provides some evidence on the clinical effects of antimicrobials and suggests they have no influence on mortality. They found that even though 196/218 (90%) of patients were given antimicrobials, none of these had a significant impact on survival, except fluoroquinolones which increased likelihood of survival (P = 0.046). Even though this p value is <0.05, interpretation of this impact should be carried out with reservation due to sampling variation and because the study had a low population (n = 218). In fact, as the sample size (n) is too small, the survival proportions observed in the sample may not be an accurate reflection of the true population proportions due to the effects of random error. Once again, there was no standardised severity scoring or treating regime as many clinicians were involved, so a broad range of clinical presentations were compared and may be confounding. The study had too many variables introduced, and the interactions of these covariates were not considered in their statistical analysis models.

Pritchard et al. (2014) studied clinical records of dogs treated for snakebite at one hospital. Reports of follow-up via phone call or email to owners were included, such as if the dog required subsequent medical care at another facility. Although no statical analysis was performed, Pritchard et al. (2014) presented data relevant to this PICO as they reported incidence of cases with signs associated with infection. Of the 52 cases reviewed, tissue necrosis at the bite site was reported by 3/27 owners (11%), which is a higher incidence than the other studies in this review. Of those, only one required follow-up care at another veterinary facility and no dog re-presented to the hospital in the study for snakebite-associated abscess formation. Nevertheless, there is no indication of whether this population had received antimicrobials, so a correlation between their use and outcome cannot be determined. In fact, only 27 cases had follow-up data, so power of study is low. Furthermore, the median duration of hospitalisation in dogs was 14.8 hours so clinicians' assessment of long-term complications is limited. The study was limited by poorly described and subjective outcomes, such as ‘whether patients either improved or worsened during their hospitalisation time’ and unreliable follow-up data such as owner assessment of infection.

Finally, the retrospective cases series by Witsil et al. (2015) looking at data from six emergency centres found that there was a significantly longer duration of hospital stay in patients that received antimicrobials (P = 0.0004). However, this may be due to the severity of the clinical picture in these cases and the finding may simply be that overall increased morbidity leads to increased likelihood of antimicrobials being used. Thus, caution is required in concluding that the longer hospital stays are directly related to antimicrobials. Despite only 64/272 (23%) of cases receiving antimicrobials, there was only a mortality of 8/272. Of these, three had been treated and five not treated with antimicrobials. This may suggest that antimicrobials have no effect on the outcome of survival, and the authors conclude that the routine use of antimicrobials was not supported by their results. Nevertheless, the paper had many limitations, for example administration of treatment was not standardised and was confounded by severity of clinical signs, clinician preference, cost of treatment and the variations between centres where the data was collected. However, the five hospitals were within a network that shared data capture methods that enhanced the standardisation of analysis. Finally, there was loss to follow-up beyond patient discharge, which causes selection bias and meant later complications following envenomation were not identified.

In summary, the findings of this review suggest that administration of antimicrobials does not have a significant effect on the incidence of wound infection, nor outcome of survival. The incidence of wound infection following envenomation in canine patients is low, which agrees with human studies, where rate of infection has been found to be 0.08% (August et al., 2018). There remain controversies around all treatment protocols used in canine patients with snakebites, as presented in the review by Armentano & Schaer (2011). This review drew comparisons between cases of human snake envenomation to advise clinicians on use of antimicrobials, but, as in this paper, they found that due to limitations of the current veterinary literature it is not possible to provide direct conclusive evidence regarding antimicrobial treatment and outcome. Armentano & Schaer (2011) recommended use of antimicrobials in snakebite cases only when there is clinical and microbiological evidence, extrapolating from human medicine where prophylactic antimicrobials are not indicated as initial therapy because of this low incidence of infection, likely due to the bactericidal effects of the venom itself. Interestingly, snakebites are considered a neglected tropical disease in human medicine and the lack of evidence supporting the guidelines for the prevention of wound infections in humans is comparable, making the topic of this review relevant from a One Health perspective. However, a Cochrane review by Bhaumik et al. (2022) is in progress; this intends to inform practice guidelines for antimicrobial treatment of snakebites in humans, the outcomes of which could potentially prove useful for extrapolation to veterinary patients.

Overall, the available evidence is weak as the studies are case series, meaning they have limited evidentiary power, have a high likelihood of bias and cannot reliably prove causation. The studies had relatively low sample sizes, meaning they all had insufficient numbers to achieve adequate power. In relation to the PICO question, these numbers are even lower if considering how many dogs did receive antimicrobials; there are insufficient cases receiving antimicrobials versus those that are not to make a significant comparison. Nevertheless, the total population of these studies was 867 dogs which is arguably a powerful sample size to analyse. At least 467 dogs overall received antimicrobials, meaning the antimicrobial treated versus untreated groups were almost proportional, which allows some clinically relevant comparisons to be made.

Proving the efficacy of treatment is problematic due to the varying degree of effects of venom in each victim and the difficulty in designing a study that can control for all treatments bar the drug of interest, without ethical issues arising. Undoubtedly, further controlled prospective studies are needed to provide better evidence-based information. Similarly, further evaluation of the incidence of wound infection in canine patients following envenomation would be informative. But until then clinicians must use their own clinical judgment from available evidence to decide on the necessary snakebite treatment for their patient.

## Methodology

**Search strategy:** 

Databases searched and dates covered:	CAB Abstracts on OVID Platform 1910–2023 Web of Science (Clarivate Analytics) 1945–2023 Scopus (Elsevier) 1960–2023
Search strategy:	CAB Abstracts: (dog* OR bitch* OR cani*) AND (antibiotic* OR antimicrobial* OR antibacterial*) AND (snake*) Web of Science: (limited to Web of Science Core Collection, Veterinary Science) (dog* OR bitch* OR cani*) AND (antibiotic* OR antimicrobial* OR antibacterial*) AND (snake*) Scopus: (limited to Veterinary subject area): (dog* OR bitch* OR cani*) AND (antibiotic* OR antimicrobial* OR antibacterial*) AND (snake*) AND (infect* OR wound* OR bite* OR envenomat*) Note the terms (infect* OR wound* OR bite* OR envenomat*) were originally included in the search strategy for CAB Abstracts and Web of Science, but yielded fewer results than the terms used above and no additional papers.
Dates searches performed:	30 Mar 2023

**Exclusion / inclusion criteria:** 

Exclusion:	Reviews of available treatments. Book chapters. Case reports. Non-English language. Papers that could not be accessed by the author or university library. Not direct analysis of antibiotic use made.
Inclusion:	Studies that cover the use of antimicrobials in canine patients bitten by snakes.

**Search outcome:** 

Database	Number of results	Excluded – Not about snakebites in dogs	Excluded – Not relevant to PICO	Excluded – No English version available	Excluded – Unable to access	Total relevant papers
CAB Abstracts	77	28	32	9	2	6
Web of Science	46	31	7	1	1	6
Scopus	316	283	23	2	4	4
Total relevant papers when duplicates removed						7

## ORCiD

Megan Ballman: https://orcid.org/0000-0002-0746-9368

Davide Messina: https://orcid.org/0000-0003-3813-5501

## Conflict of interest

The authors declare no conflicts of interest. Megan Ballman would like to thank Martin Hawes for their support in this work.
